# Role of the Aryl Hydrocarbon Receptor in Colon Neoplasia

**DOI:** 10.3390/cancers7030847

**Published:** 2015-07-31

**Authors:** Guofeng Xie, Jean-Pierre Raufman

**Affiliations:** Division of Gastroenterology and Hepatology, Veterans Administration Maryland Health Care System, University of Maryland School of Medicine, Baltimore, MD 21201, USA; E-Mail: jraufman@medicine.umaryland.edu

**Keywords:** colorectal cancer, TCDD, aryl hydrocarbon receptor, xenobiotics

## Abstract

For both men and women, colorectal cancer (CRC) is the second leading cause of cancer death in the United States, primarily as a consequence of limited therapies for metastatic disease. The aryl hydrocarbon receptor (AhR) is a ligand-dependent transcription factor with diverse functions in detoxification of xenobiotics, inflammatory responses, and tissue homeostasis. Emerging evidence indicates that AhR also plays an important role in regulating intestinal cell proliferation and tumorigenesis. Here, we review both the pro- and anti-carcinogenic properties of AhR signaling and its potential role as a therapeutic target in CRC.

## 1. Introduction

### 1.1. Colorectal Cancer is a Major Health Concern

In the U.S., Colorectal cancer (CRC) is the second leading cause of cancer death in men and women combined [1,2,3,4]. The American Cancer Society estimates that in 2015, 137,000 people will be diagnosed with CRC, 50,000 of whom will die from this disease [5]. Although colonoscopy and stool-based screening tests are effective at preventing and reducing mortality from CRC, many people fail to undergo such tests [6]. Also, the efficacy of colon cancer screening is limited by the sensitivity of stool-based tests and “miss” rates on colonoscopy [7,8]. Non-steroidal anti-inflammatory drugs are marginally effective chemo-preventive agents [9,10] and limited by gastrointestinal [11] and cardiovascular [12] toxicity that led to the withdrawal of rofecoxib [13]. Chemotherapy and radiation are not very effective for patients with advanced disease. Recently, the use of biologicals targeting vascular endothelial growth factor (VEGF) and the epidermal growth factor receptor (EGFR; *i.e.*, Bevacizumab, Cetuximab and Panitumumab) were shown to increase survival in patients with advanced CRC, but only by a few months with limited impact on five-year survival—which remains on the order of 5–10% [14,15,16]. Furthermore, the use of these agents is limited by off-target toxicity that includes gastrointestinal bleeding and skin rashes that often lead to discontinuation of therapy [17].

### 1.2. The Aryl Hydrocarbon Receptor

The aryl hydrocarbon receptor (AhR) is a cytosolic ligand-dependent transcription factor that belongs to the basic helix-loop-helix/Per-Arnt-Sim (bHLH/PAS) family [18]. AhR has diverse physiological functions including detoxification of environmental pollutants, mucosal inflammation and tissue regeneration [19]. When inactive, AhR remains bound to several chaperon proteins including heat-shock protein 90 and the AhR-interacting protein [20,21,22,23]. Upon ligand binding, cytosolic AhR translocates into the nucleus and forms a heterodimer with the AhR nuclear translocator (ARNT) protein, which then interacts with dioxin responsive elements (DREs) on the promoters of AhR target genes including cytochrome P450 enzymes CYP1A1/A2/B1, thereby activating transcription [20]. 

### 1.3. AhR Ligands

Numerous AhR ligands have been identified, including synthetic and environmental chemicals, naturally-occurring dietary and endogenous compounds [24,25,26,27]. Environmental pollutants include planar halogenated aromatics [e.g., 2,3,7,8-tetrachlorodibenzo-*p*-dioxin (TCDD)], synthetic polynuclear aromatic hydrocarbons and dioxin-like compounds. TCDD is the prototype of many chlorinated aromatic compounds that are abundant contaminants in the environment. With low-dose administration to rodents, TCDD acts as a carcinogen and causes tumors in multiple sites including the liver, lung, nasal turbinates, hard palate, thyroid, tongue, and skin, but not the intestines [28,29], indicating tissue-specific carcinogenic properties. Other common toxicants include halogenated dibenzo-*p*-dioxins, dibenzofurans, azo(xy)benzenes, and naphthalenes. Manufactured drugs include proton pump inhibitors, Sulindac and 4-hydroxytamoxifen. Candidates for endogenous ligands include indigoids and metabolites of arachidonic acid, heme and tryptophan. AhR antagonists include CH223191, GNF351, and 3ʹ-4ʹ-Dimethoxy-α-Naphthoflavone [25].

Colorectal cancer is often linked to a “so-called” Western diet, rich in carbohydrates, red meats and saturated fats, but low in vegetables and fresh fruits [30,31]. Recent data have shown that certain dietary components from vegetables have protective effects against CRC [32]. Several natural dietary AhR ligands may play important roles in mediating this protective effect. The tryptophan metabolites indole derivatives, including indole-3-acetic acid (IAA) and indole-3-carbinol (I3C) are natural AhR ligands generated by conversion from dietary tryptophan. Another indole derivative, 3,3ʹ-diindolylmethane (DIM) is converted from glucosinolates by commensal intestinal microbiota [33]. Glucosinolates are natural components of many pungent plants and cruciferous vegetables such as mustard, cabbage, cauliflower, broccoli and horseradish. An I3C derivative indole[3,2-*b*]carbazole (ICZ) has strong receptor binding affinity for AhR. It is reported that glucosinolates and tryptophan metabolites have chemo-preventive effects on CRC [34,35,36], perhaps by enhancing apoptosis in cancer cells [34] or modulating gut immune homeostasis [37,38].

Naturally-occurring flavonoids, ubiquitous in many fruits and vegetables, are another class of dietary AhR ligands that have chemo-preventive effects for CRC [39]. Statistically significant reductions in CRC risk were associated with the five classes of flavonoids-flavonols, quercetin, catechin, epicatechin and procyanisins. Risk reductions were greater in non-smokers. Many of these flavonoids activate AhR *in vitro* [40,41]. Veeriah *et al.* reported that flavonoids extracted from apples inhibited growth (cell proliferation) of HT29 human CRC cells [42].

## 2. Role of AhR in Colon Tumorigenesis

### 2.1. AhR Is a Tumor Suppressor in Mouse Models of CRC

The role of AhR in carcinogenesis remains controversial. Recent evidence supports both pro- and anti-carcinogenic properties of AhR signaling, perhaps in a tissue-selective manner. The Wnt/β-catenin signaling is a major signal transduction pathway involved in colon carcinogenesis. In quiescent cells β-catenin is sequestered in a multi-protein complex, including axin, adenomatous polyposis coli (APC) and glycogen synthase kinase-3β (GSK-3β), that targets β-catenin for phosphorylation, ubiquitination and proteosomal degradation [43,44]. Wnt ligands activate a cascade that inhibits GSK-3β-induced β-catenin phosphorylation, frees β-catenin from the destruction complex, and allows its nuclear translocation and subsequent activation of target genes leading to increased cell proliferation and tumorigenesis [43,44,45]. Kawajiri *et al.* showed that AhR-deficient mice spontaneously develop cecal adenocarcinomas by the age of 30 to 40 weeks [46]. These investigators demonstrated dual roles for AhR in regulating intracellular protein levels, both as a ligand-activated transcription factor and as a ligand-dependent E3 ubiquitin ligase [18,46]. AhR suppresses intestinal carcinogenesis by a ligand-dependent β-catenin degradation pathway that functions independently of and cooperatively with the canonical APC-dependent system. Natural AhR ligands converted from dietary tryptophan and glucoinolates in the intestines are as efficient as exogenous xenobiotic ligands in suppressing tumor formation in *Apc^min/+^* mice [46]. In addition, AhR also functions as a tumor suppressor for liver carcinogenesis by inhibiting cell proliferation through G0-G1 cell cycle arrest [47]. 

### 2.2. AhR Expression in Colon Tumors

AhR is ubiquitously expressed in mouse and human tissues, including the gastrointestinal tract [48,49]. The AhR mRNA expression profile of 967 human cancer cell lines showed that moderate levels of AhR are expressed in colon cancer cells [50]. It is not clear how this level of AhR expression compares to that of normal intestinal epithelial cells. Interestingly, reduced AhR expression was observed in specimens of human cecal cancers and adjacent tissues [46]. 

### 2.3. AhR Target Genes and Their Roles in CRC

AhR may regulate intestinal tumorigenesis through its target genes, including the Phase I drug metabolizing enzymes CYP1A1, CYP1A2, and CYP1B1. CYP1A1 and CYP1B1 are extrahepatic enzymes that catalyze conversion of polycyclic aromatic hydrocarbons including benzo[a]pyrene to active genotoxic metabolites, thereby contributing to carcinogenesis [51]. Androutsopoulos *et al.* showed that CYP1A1 and CYP1B1 are overexpressed in 80% and 60% of human colon tumors, respectively, suggesting an important role for these enzymes in colon neoplasia [52]. In addition, it was shown that AhR activation in colon cancer cells induces expression of multiple target genes including matrix metalloproteinase (MMP)-9, calcium ion flux, pro-inflammatory IL-1β and the drug transporter BCRP/ABCG2 [53,54,55]. 

### 2.4. AhR Cross-Talks with Multiple Signaling Pathways

**Figure 1:**
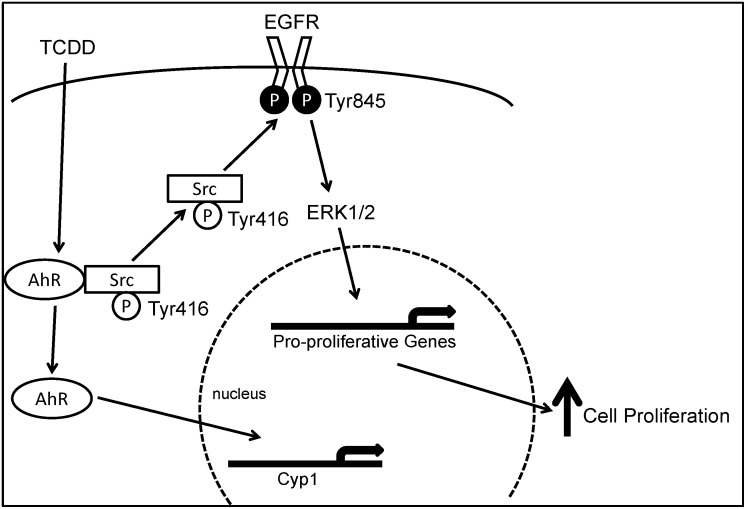
Model depicting molecular mechanisms underlying 2,3,7,8-tetrachlorodibenzo-*p*-dioxin (TCDD)-induced, Src-mediated activation of the epidermal growth factor receptor (EGFR) signaling in colon epithelial cells. Binding of aryl hydrocarbon receptor (AhR) by ligand, e.g., TCDD, results in phosphorylation of Src kinase at Tyr416 and de-phosphorylation of Src kinase at Tyr527. Activated Src either directly phosphorylates EGFR at Tyr 485 or indirectly activate EGFR through matrix metalloproteinase (MMP) and heparin-binding (HB)EGF, leading to activation of EGFR and downstream ERK1/2 signaling, which stimulates gene transcription and cell proliferation. Binding of AhR by TCDD also causes translocation of AhR from the cytosol to the nucleus where it acts as a transcription factor for *CYP1A1* and *CYP1B1*. Reproduced with permission from G. Xie, Z. Peng and J.P. Raufman. *Am. J. Physiol. Gastrointest. Liver Physiol.*; published by the American Physiological Society, 2012.

AhR cross-talks with multiple growth factor-mediated signal transduction pathways including transforming growth factor-β, tumor necrosis factor-α, EGFR and Src pathways [56,57,58,59,60]. As shown in Figure 1, we demonstrated that in human colon cancer cell lines, upon AhR activation by TCDD, Src-mediated cross-talk between AhR and EGFR results in ERK1/2 activation and enhanced cell proliferation [61]. Also, AhR interacts with the retinoblastoma protein (pRB) protein to inhibit G1 to S phase cell cycle transition via protein kinase C and p38 MAPK [62].

### 2.5. Role of AhR in Chronic Intestinal Inflammation

AhR activation by dietary ligands is required for the maintenance of intestinal immune homeostasis including innate immune cells such as intraepithelial lymphocytes (IELs) and interleukin (IL)-22-producing lymphoid cells [63]. AhR-deficient mice lack IELs and are more susceptible to bacterial infections and experimental colitis. In patients with Crohn’s disease, intestinal T cells and natural killer cells have reduced AhR expression levels [64]. Furumatsu *et al.* showed that dextran sodium sulfate (DSS)-induced colitis was more severe in AhR-knockout mice than in wild-type mice [65]. Administration of DSS increased AhR expression in the colonic epithelium [65]. Also, oral administration of the AhR agonist β-naphthoflavone attenuated DSS-evoked colitis [65]. In addition, AhR activation by 6-formylindolo (3,2-b) carbazole (Ficz) down-regulates IL-7 and reduces inflammation in DSS-induced colitis [66].

### 2.6. Role of AhR in Inflammation-Associated Colon Neoplasia

The risk of CRC is increased with chronic intestinal inflammation as is observed with inflammatory bowel disease, including both Crohn’s and ulcerative colitis [67]. Ikuta *et al.* showed that AhR-deficient mice develop cecal tumors with severe inflammation which is dependent on the apoptosis-associated speck-like protein containing a caspase recruitment protein (ASC) [68]. In AhR-deficient mice, blocking interleukin (IL)-1β signaling with a caspase-1 inhibitor attenuated cecal tumorigenesis in AhR-deficient mice [68]. Also, germ-free AhR (−/−) and AhR (−/−)/ASC (−/−) mice had reduced tumor formation compared with AhR (−/−) mice [68]. These observations suggest that AhR also acts as tumor suppressor in inflammation-associated intestinal neoplasia.

### 2.7. Role of AhR in Circadian Clock Circuitry and CRC

Growing evidence indicates that there is physiological cross-talk between AhR and the circadian system [69]. The AhR complex is involved in sensing and transforming environmental xenobiotics and naturally occurring AhR ligands. Epidemiological studies showed an increased incidences of CRC in shift workers, suggesting disruption of circadian timing may contribute to CRC carcinogenesis, possibly by interrupting intestinal homeostatic control of cell proliferation, differentiation and apoptosis [70].

## 3. Conclusions

Emerging evidence suggests that AhR and its ligands play important roles in intestinal tumorigenesis, identifying AhR as a potential therapeutic target for CRC. Although most previously-known AhR ligands are toxic HAs and genotoxic PAHs, many newly-identified AhR agonists are non-toxic and have therapeutic potential [24,25,26]. However, there remain inconsistencies regarding the role of AhR in colon neoplasia. Studies in animal models of CRC suggest that in the basal state without xenobiotic ligands AhR acts as a tumor suppressor, particularly in cecal tumors. This tumor-suppressive action of AhR is radically different from the pro-oncogenic role that AhR plays as a receptor for pro-carcinogenic ligands such as TCDD. Over-expression of CYP1A1 and CYP1B1 in human colon tumors implies a pro-carcinogenic role for AhR target genes. In addition, studies using human colon cancer cell lines showed that AhR activation *in vitro* increases cell proliferation, migration, and invasion via transactivation of the EGFR/ERK signaling pathway. Interestingly, most *in vivo* studies demonstrated protective effects of AhR in CRC; in contrast, many *in vitro* studies showed pro-carcinogenic effects of AhR ligands. This disconnect suggests that colon cancer cell lines may not always be reliable for predicting the *in vivo* role of AhR in colon carcinogenesis. Further translational studies are needed to clarify the role of AhR in different stages of CRC progression, including initiation, growth and dissemination. Such information will be useful to guide potential AhR-based therapies for CRC. 

## References

[1] Siegel R., Desantis C., Jemal A. (2014). Colorectal cancer statistics, 2014. CA Cancer J. Clin..

[2] Peery A.F., Dellon E.S., Lund J., Crockett S.D., McGowan C.E., Bulsiewicz W.J., Gangarosa L.M., Thiny M.T., Stizenberg K., Morgan D.R. (2012). Burden of gastrointestinal disease in the united states: 2012 update. Gastroenterology.

[3] Edwards B.K., Ward E., Kohler B.A., Eheman C., Zauber A.G., Anderson R.N., Jemal A., Schymura M.J., Lansdorp-Vogelaar I., Seeff L.C. (2010). Annual report to the nation on the status of cancer, 1975–2006, featuring colorectal cancer trends and impact of interventions (risk factors, screening, and treatment) to reduce future rates. Cancer.

[4] Jemal A., Siegel R., Xu J., Ward E. (2010). Cancer statistics, 2010. CA Cancer J. Clin..

[5] Siegel R.L., Miller K.D., Jemal A. (2015). Cancer statistics, 2015. CA Cancer J. Clin..

[6] Hewett D.G., Rex D.K. (2015). The big picture: Does colonoscopy work?. Gastrointest. Endosc. Clin. N. Am..

[7] Pignone M., Saha S., Hoerger T., Mandelblatt J. (2002). Cost-effectiveness analyses of colorectal cancer screening: A systematic review for the U.S. Preventive services task force. Ann. Intern. Med..

[8] Corley D.A. (2011). The future of colon cancer screening: What do we recommend and will it be too much, too little, or just right?. Gastroenterology.

[9] Baron J.A., Sandler R.S., Bresalier R.S., Quan H., Riddell R., Lanas A., Bolognese J.A., Oxenius B., Horgan K., Loftus S. (2006). A randomized trial of rofecoxib for the chemoprevention of colorectal adenomas. Gastroenterology.

[10] Bertagnolli M.M., Eagle C.J., Zauber A.G., Redston M., Solomon S.D., Kim K., Tang J., Rosenstein R.B., Wittes J., Corle D. (2006). Celecoxib for the prevention of sporadic colorectal adenomas. N. Engl. J. Med..

[11] Helin-Salmivaara A., Saarelainen S., Gronroos J.M., Vesalainen R., Klaukka T., Huupponen R. (2007). Risk of upper gastrointestinal events with the use of various nsaids: A case-control study in a general population. Scand. J. Gastroenterol..

[12] Kerr D.J., Dunn J.A., Langman M.J., Smith J.L., Midgley R.S., Stanley A., Stokes J.C., Julier P., Iveson C., Duvvuri R. (2007). Rofecoxib and cardiovascular adverse events in adjuvant treatment of colorectal cancer. N. Engl. J. Med..

[13] McGettigan P., Henry D. (2006). Cardiovascular risk and inhibition of cyclooxygenase: A systematic review of the observational studies of selective and nonselective inhibitors of cyclooxygenase 2. JAMA.

[14] Modjtahedi H., Essapen S. (2009). Epidermal growth factor receptor inhibitors in cancer treatment: Advances, challenges and opportunities. Anticancer Drugs.

[15] Overman M.J., Hoff P.M. (2007). Egfr-targeted therapies in colorectal cancer. Dis. Colon Rectum.

[16] Venook A.P. (2005). Epidermal growth factor receptor-targeted treatment for advanced colorectal carcinoma. Cancer.

[17] Sipples R. (2006). Common side effects of anti-egfr therapy: Acneform rash. Semin. Oncol. Nurs..

[18] Ohtake F., Fujii-Kuriyama Y., Kato S. (2009). AhR acts as an E3 ubiquitin ligase to modulate steroid receptor functions. Biochem. Pharmacol..

[19] Adachi Y., Yamamoto H., Itoh F., Hinoda Y., Okada Y., Imai K. (1999). Contribution of matrilysin (MMP-7) to the metastatic pathway of human colorectal cancers. Gut.

[20] Hankinson O. (1995). The aryl hydrocarbon receptor complex. Annu. Rev. Pharmacol. Toxicol..

[21] Carver L.A., Bradfield C.A. (1997). Ligand-dependent interaction of the aryl hydrocarbon receptor with a novel immunophilin homolog *in vivo*. J. Biol. Chem..

[22] Chen H.S., Perdew G.H. (1994). Subunit composition of the heteromeric cytosolic aryl hydrocarbon receptor complex. J. Biol. Chem..

[23] Ma Q., Whitlock J.P. (1997). A novel cytoplasmic protein that interacts with the ah receptor, contains tetratricopeptide repeat motifs, and augments the transcriptional response to 2,3,7,8-tetrachlorodibenzo-*p*-dioxin. J. Biol. Chem..

[24] Denison M.S., Nagy S.R. (2003). Activation of the aryl hydrocarbon receptor by structurally diverse exogenous and endogenous chemicals. Annu. Rev. Pharmacol. Toxicol..

[25] Nguyen L.P., Bradfield C.A. (2008). The search for endogenous activators of the aryl hydrocarbon receptor. Chem. Res. Toxicol..

[26] Safe S., Lee S.O., Jin U.H. (2013). Role of the aryl hydrocarbon receptor in carcinogenesis and potential as a drug target. Toxicol. Sci..

[27] Murray I.A., Patterson A.D., Perdew G.H. (2014). Aryl hydrocarbon receptor ligands in cancer: Friend and foe. Nat. Rev. Cancer.

[28] Bock K.W., Kohle C. (2005). Ah receptor- and TCDD-mediated liver tumor promotion: Clonal selection and expansion of cells evading growth arrest and apoptosis. Biochem. Pharmacol..

[29] Knerr S., Schrenk D. (2006). Carcinogenicity of 2,3,7,8-tetrachlorodibenzo-*p*-dioxin in experimental models. Mol. Nutr. Food Res..

[30] Chao A., Thun M.J., Connell C.J., McCullough M.L., Jacobs E.J., Flanders W.D., Rodriguez C., Sinha R., Calle E.E. (2005). Meat consumption and risk of colorectal cancer. JAMA.

[31] Kirkegaard H., Johnsen N.F., Christensen J., Frederiksen K., Overvad K., Tjonneland A. (2010). Association of adherence to lifestyle recommendations and risk of colorectal cancer: A prospective danish cohort study. BMJ.

[32] Louis P., Hold G.L., Flint H.J. (2014). The gut microbiota, bacterial metabolites and colorectal cancer. Nat. Rev. Microbiol..

[33] Bjeldanes L.F., Kim J.Y., Grose K.R., Bartholomew J.C., Bradfield C.A. (1991). Aromatic hydrocarbon responsiveness-receptor agonists generated from indole-3-carbinol *in vitro* and *in vivo*: Comparisons with 2,3,7,8-tetrachlorodibenzo-*p*-dioxin. Proc. Natl. Acad. Sci. USA.

[34] Bonnesen C., Eggleston I.M., Hayes J.D. (2001). Dietary indoles and isothiocyanates that are generated from cruciferous vegetables can both stimulate apoptosis and confer protection against DNA damage in human colon cell lines. Cancer Res..

[35] Kim Y.S., Milner J.A. (2005). Targets for indole-3-carbinol in cancer prevention. J. Nutr. Biochem..

[36] Potter J.D., Steinmetz K. (1996). Vegetables, fruit and phytoestrogens as preventive agents. IARC Sci. Publ..

[37] Jin U.H., Lee S.O., Sridharan G., Lee K., Davidson L.A., Jayaraman A., Chapkin R.S., Alaniz R., Safe S. (2014). Microbiome-derived tryptophan metabolites and their aryl hydrocarbon receptor-dependent agonist and antagonist activities. Mol. Pharmacol..

[38] Zelante T., Iannitti R.G., Cunha C., de Luca A., Giovannini G., Pieraccini G., Zecchi R., D’Angelo C., Massi-Benedetti C., Fallarino F. (2013). Tryptophan catabolites from microbiota engage aryl hydrocarbon receptor and balance mucosal reactivity via interleukin-22. Immunity.

[39] Theodoratou E., Kyle J., Cetnarskyj R., Farrington S.M., Tenesa A., Barnetson R., Porteous M., Dunlop M., Campbell H. (2007). Dietary flavonoids and the risk of colorectal cancer. Cancer Epidemiol. Biomarkers Prev..

[40] Amakura Y., Tsutsumi T., Nakamura M., Kitagawa H., Fujino J., Sasaki K., Toyoda M., Yoshida T., Maitani T. (2003). Activation of the aryl hydrocarbon receptor by some vegetable constituents determined using *in vitro* reporter gene assay. Biol. Pharm. Bull..

[41] Zhang S., Qin C., Safe S.H. (2003). Flavonoids as aryl hydrocarbon receptor agonists/antagonists: Effects of structure and cell context. Environ. Health Perspect..

[42] Veeriah S., Kautenburger T., Habermann N., Sauer J., Dietrich H., Will F., Pool-Zobel B.L. (2006). Apple flavonoids inhibit growth of HT29 human colon cancer cells and modulate expression of genes involved in the biotransformation of xenobiotics. Mol. Carcinog..

[43] Moon R.T., Kohn A.D., de Ferrari G.V., Kaykas A. (2004). Wnt and beta-catenin signalling: Diseases and therapies. Nat. Rev. Genet..

[44] Clevers H. (2006). Wnt/beta-catenin signaling in development and disease. Cell.

[45] Markowitz S.D., Bertagnolli M.M. (2009). Molecular origins of cancer: Molecular basis of colorectal cancer. N. Engl. J. Med..

[46] Kawajiri K., Kobayashi Y., Ohtake F., Ikuta T., Matsushima Y., Mimura J., Pettersson S., Pollenz R.S., Sakaki T., Hirokawa T. (2009). Aryl hydrocarbon receptor suppresses intestinal carcinogenesis in apcmin/+ mice with natural ligands. Proc. Natl. Acad. Sci. USA.

[47] Fan Y., Boivin G.P., Knudsen E.S., Nebert D.W., Xia Y., Puga A. (2010). The aryl hydrocarbon receptor functions as a tumor suppressor of liver carcinogenesis. Cancer Res..

[48] Dolwick K.M., Schmidt J.V., Carver L.A., Swanson H.I., Bradfield C.A. (1993). Cloning and expression of a human ah receptor cdna. Mol. Pharmacol..

[49] Hayashi S., Watanabe J., Nakachi K., Eguchi H., Gotoh O., Kawajiri K. (1994). Interindividual difference in expression of human ah receptor and related p450 genes. Carcinogenesis.

[50] O’Donnell E.F., Kopparapu P.R., Koch D.C., Jang H.S., Phillips J.L., Tanguay R.L., Kerkvliet N.I., Kolluri S.K. (2012). The aryl hydrocarbon receptor mediates leflunomide-induced growth inhibition of melanoma cells. PLoS ONE.

[51] Androutsopoulos V.P., Tsatsakis A.M., Spandidos D.A. (2009). Cytochrome p450 cyp1a1: Wider roles in cancer progression and prevention. BMC Cancer.

[52] Androutsopoulos V.P., Spyrou I., Ploumidis A., Papalampros A.E., Kyriakakis M., Delakas D., Spandidos D.A., Tsatsakis A.M. (2013). Expression profile of cyp1a1 and cyp1b1 enzymes in colon and bladder tumors. PLoS ONE.

[53] Le Ferrec E., Lagadic-Gossmann D., Rauch C., Bardiau C., Maheo K., Massiere F., le Vee M., Guillouzo A., Morel F. (2002). Transcriptional induction of cyp1a1 by oltipraz in human caco-2 cells is aryl hydrocarbon receptor- and calcium-dependent. J. Biol. Chem..

[54] Tompkins L.M., Li H., Li L., Lynch C., Xie Y., Nakanishi T., Ross D.D., Wang H. (2010). A novel xenobiotic responsive element regulated by aryl hydrocarbon receptor is involved in the induction of BCRP/ABCG2 in LS174T cells. Biochem. Pharmacol..

[55] Villard P.H., Caverni S., Baanannou A., Khalil A., Martin P.G., Penel C., Pineau T., Seree E., Barra Y. (2007). PPARalpha transcriptionally induces ahr expression in Caco-2, but represses AhR pro-inflammatory effects. Biochem. Biophys. Res. Commun..

[56] Haarmann-Stemmann T., Bothe H., Abel J. (2009). Growth factors, cytokines and their receptors as downstream targets of arylhydrocarbon receptor (AhR) signaling pathways. Biochem. Pharmacol..

[57] Puga A., Ma C., Marlowe J.L. (2009). The aryl hydrocarbon receptor cross-talks with multiple signal transduction pathways. Biochem. Pharmacol..

[58] Gomez-Duran A., Carvajal-Gonzalez J.M., Mulero-Navarro S., Santiago-Josefat B., Puga A., Fernandez-Salguero P.M. (2009). Fitting a xenobiotic receptor into cell homeostasis: How the dioxin receptor interacts with tgfbeta signaling. Biochem. Pharmacol..

[59] De Oliveira S.K., Smolenski A. (2009). Phosphodiesterases link the aryl hydrocarbon receptor complex to cyclic nucleotide signaling. Biochem. Pharmacol..

[60] Rowlands J.C., Gustafsson J.A. (1997). Aryl hydrocarbon receptor-mediated signal transduction. Crit. Rev. Toxicol..

[61] Xie G., Peng Z., Raufman J.P. (2012). Src-mediated aryl hydrocarbon and epidermal growth factor receptor cross talk stimulates colon cancer cell proliferation. Am. J. Physiol. Gastrointest. Liver Physiol..

[62] Dietrich C., Kaina B. (2010). The aryl hydrocarbon receptor (AhR) in the regulation of cell-cell contact and tumor growth. Carcinogenesis.

[63] Monteleone I., MacDonald T.T., Pallone F., Monteleone G. (2012). The aryl hydrocarbon receptor in inflammatory bowel disease: Linking the environment to disease pathogenesis. Curr. Opin. Gastroenterol..

[64] Monteleone I., Rizzo A., Sarra M., Sica G., Sileri P., Biancone L., MacDonald T.T., Pallone F., Monteleone G. (2011). Aryl hydrocarbon receptor-induced signals up-regulate il-22 production and inhibit inflammation in the gastrointestinal tract. Gastroenterology.

[65] Furumatsu K., Nishiumi S., Kawano Y., Ooi M., Yoshie T., Shiomi Y., Kutsumi H., Ashida H., Fujii-Kuriyama Y., Azuma T. (2011). A role of the aryl hydrocarbon receptor in attenuation of colitis. Dig. Dis. Sci..

[66] Ji T., Xu C., Sun L., Yu M., Peng K., Qiu Y., Xiao W., Yang H. (2015). Aryl hydrocarbon receptor activation down-regulates IL-7 and reduces inflammation in a mouse model of DSS-induced colitis. Dig. Dis. Sci..

[67] Ullman T.A., Itzkowitz S.H. (2011). Intestinal inflammation and cancer. Gastroenterology.

[68] Ikuta T., Kobayashi Y., Kitazawa M., Shiizaki K., Itano N., Noda T., Pettersson S., Poellinger L., Fujii-Kuriyama Y., Taniguchi S. (2013). Asc-associated inflammation promotes cecal tumorigenesis in aryl hydrocarbon receptor-deficient mice. Carcinogenesis.

[69] Anderson G., Beischlag T.V., Vinciguerra M., Mazzoccoli G. (2013). The circadian clock circuitry and the AhR signaling pathway in physiology and pathology. Biochem. Pharmacol..

[70] Mazzoccoli G., Vinciguerra M., Papa G., Piepoli A. (2014). Circadian clock circuitry in colorectal cancer. World J. Gastroenterol..

